# IMPLEMENTING SMART AMBIENT BRIGHT LIGHT FOR NURSING HOME RESIDENTS WITH DEMENTIA: ASSESSING FIDELITY

**DOI:** 10.1093/geroni/igae098.4349

**Published:** 2024-12-31

**Authors:** Diane Berish, Julian Wang, Shevvaa Beiglary, Ying-Ling Jao, Yo-Jen Liao

**Affiliations:** The Pennsylvania State University, University Park, Pennsylvania, United States; Pennsylvania State University, University Park, Pennsylvania, United States; Penn State University, University Park, Pennsylvania, United States; Pennsylvania State University, University Park, Pennsylvania, United States; The Pennsylvania State University, University Park, Pennsylvania, United States

## Abstract

Up to 90% of persons with dementia experience neurobehavioral symptoms and poor sleep which may be associated with limited daylight exposure and circadian disruptions. Lighting interventions regulate the circadian rhythm and have been shown to reduce agitation. Smart Ambient Bright Light (SABL) incorporates natural light and is an automatic lighting system that delivers bright light during the day (photopic illuminance ≥400 lux, circadian stimulus [CS]≥0.3) and dim light during the night (lux≤40, CS ≤0.1). Two methods were used to assess SABL fidelity. First, manual measurements were conducted during the daytime twice a week. Second, each participant wore a light sensor to measure individual lighting exposure for 24 hours, 7 days a week, every other week. Manual measurement results indicated that the SABL lighting conditions across different spaces generally met the targets during daytime (lux: mean=394.2, SD=49.75; CS: mean=0.302, SD=0.07). Individual sensors also showed that participants’ average daytime light exposure during the intervention met the target for lux (mean=406.9, CI=369.9-443.8) and CS (mean=0.363, CI= 0.332-0.393). Individual nighttime lighting exposure during the intervention was above target for lux (mean=94.5. CI= 82.3-106.7) and CS (mean=0.111, CI=0.098-0.124). Additionally, participants received higher daytime CS (t=-8.7, p< 0.001) and daytime lux (t=-8.48, p< 0.0001) while also receiving lower nighttime CS (t=4.43, p=0.0001) and nighttime lux (t=7.4, p< 0.0001) during the intervention period than the control period. The results indicate the SABL achieved fidelity for daytime, but its fidelity for nighttime, especially lux, could be improved. Future studies should incorporate lessons learned to improve SABL implementation fidelity.

